# Cerebral Air Embolism from Angioinvasive Cavitary Aspergillosis

**DOI:** 10.1155/2014/406106

**Published:** 2014-08-14

**Authors:** Chen Lin, George A. Barrio, Lynne M. Hurwitz, Peter G. Kranz

**Affiliations:** ^1^Department of Neurology, Duke University, Durham, NC 27710, USA; ^2^Department of Radiology, Duke University, Durham, NC 27710, USA

## Abstract

*Background*. Nontraumatic cerebral air embolism cases are rare. We report a case of an air embolism resulting in cerebral infarction related to angioinvasive cavitary aspergillosis. To our knowledge, there have been no previous reports associating these two conditions together. *Case Presentation*. A 32-year-old female was admitted for treatment of acute lymphoblastic leukemia (ALL). Her hospital course was complicated by pulmonary aspergillosis. On hospital day 55, she acutely developed severe global aphasia with right hemiplegia. A CT and CT-angiogram of her head and neck were obtained demonstrating intravascular air emboli within the left middle cerebral artery (MCA) branches. She was emergently taken for hyperbaric oxygen therapy (HBOT). Evaluation for origin of the air embolus revealed an air focus along the left lower pulmonary vein. Over the course of 48 hours, her symptoms significantly improved. *Conclusion*. This unique case details an immunocompromised patient with pulmonary aspergillosis cavitary lesions that invaded into a pulmonary vein and caused a cerebral air embolism. With cerebral air embolisms, the acute treatment option differs from the typical ischemic stroke pathway and the provider should consider emergent HBOT. This case highlights the importance of considering atypical causes of acute ischemic stroke.

## 1. Background

Arterial air embolisms are relatively uncommon complications that can cause ischemia in any organ system. For air embolisms to occur, two situations need to be present: (1) a communication between the air and the vasculature and (2) pressure gradient favoring the passage of air into the circulation. The etiologies typically result from surgical or traumatic mechanisms (intravascular catheterization, pulmonary barotraumas, and endoscopic procedures) or decompression sickness [1–3]. Cerebral air embolisms are more rare. These have been associated mostly with intravascular catheterization and pulmonary barotrauma/mechanical ventilation [4, 5]. We report a case of cerebral air embolism caused by a cavitary aspergillus lesion. On review of the literature, there have been no previous reports associating these two conditions together without other inciting factors such as mechanical ventilation or cardiopulmonary resuscitation [6].

## 2. Case Report

A 32-year-old right-handed female with T-cell ALL admitted to the hematology service for chemotherapy and anticipating cord blood transplant. She had a complicated medical course after chemotherapy including admissions into the Medical ICU for septic shock, febrile neutropenia, acute hypoxic respiratory failure, clostridium perfringens bacteremia, persistent vancomycin resistant enterococcus (VRE) bacteremia, and pulmonary aspergillosis. The diagnosis of aspergillosis was made on hospital day 33 with CT-chest findings of a diffuse severe pneumonia with a positive serum Galactomannan EIA confirmed twice. Repeated chest radiographs revealed the development of a cavitary lesion, a radiographic finding that can be seen with aspergillosis. She was started on voriconazole and followed by the transplant infectious disease service for therapeutic voriconazole levels. She also underwent evaluation for endocarditis with a normal transthoracic echocardiogram on hospital day 35 and normal cardiac MRI on hospital day 44. She was unable to safely undergo a transesophageal echocardiogram due to a persistently low platelet count. Hospital day 45 was the last positive blood culture for her persistent VRE bacteremia with subsequent negative blood cultures. She had no invasive procedures in the week prior to the event described below.

**Figure 1:**
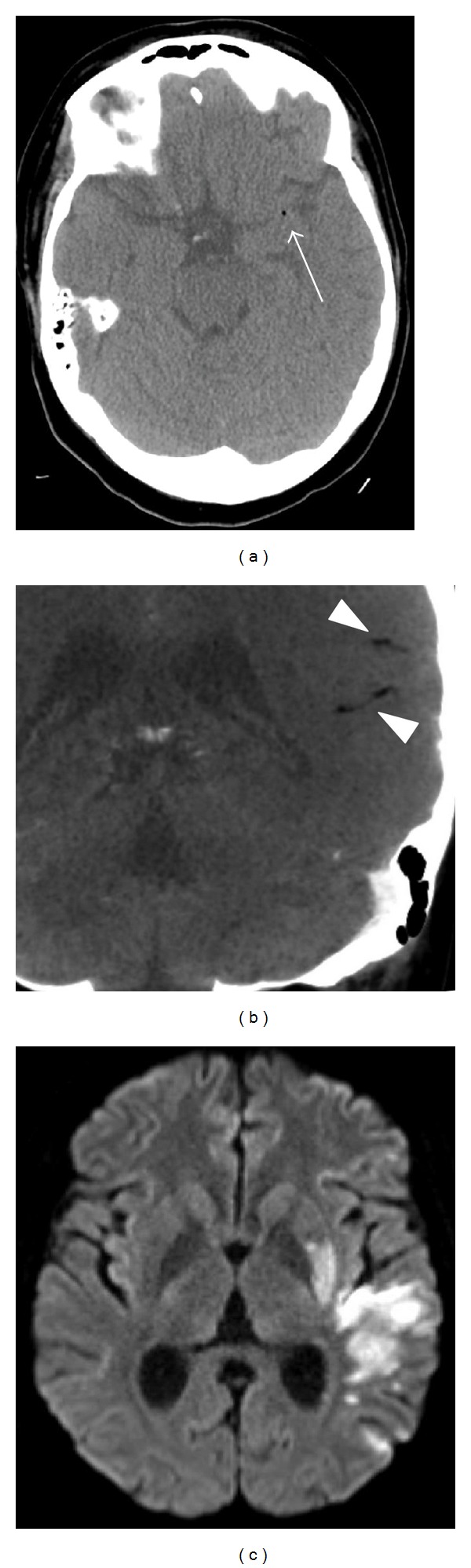
Air emboli on CT (a), CTA (b), and infarction on DWI sequence of MRI (c).

On the morning of hospital day 55, a normal neurologic exam was noted at 9 am. At 9:40 am, the patient rose from bed, felt dizzy, and slid to the floor. She was initially able to explain that she felt weak. Nursing noted no jerking movements of her extremities or bowel or urinary incontinence. Capillary blood glucose and vitals were unremarkable with glucose of 160, pulse 90 s, BP 120 s/60 s, and O_2_ 96% on room air. When primary team arrived, they noted a right facial droop, severe aphasia, with right upper and right lower extremity paresis. They called a stroke code at 9:53 am with neurology evaluation starting at 9:58 am. Her initial exam confirmed a global aphasia and right-sided hemiplegia. Her initial NIH stroke scale was 18. She was taken for emergent CT and CT-Angiography of the head and neck. She was found to have foci of intravascular air within the distal portion of the M1 segment of the left MCA, as well as additional foci of intravascular air in the branches of the inferior division of the left MCA (Figure 1). Hyperbaric service was emergently contacted to get her treatment with eventual transport to chamber by 6 pm. She completed a US Navy Treatment Table 6 protocol with no significant difficulties. After the HBOT, she had improvement of motor strength in her right arm. Her speech was spontaneous but she provided inappropriate words for the questions asked.

**Figure 2:**
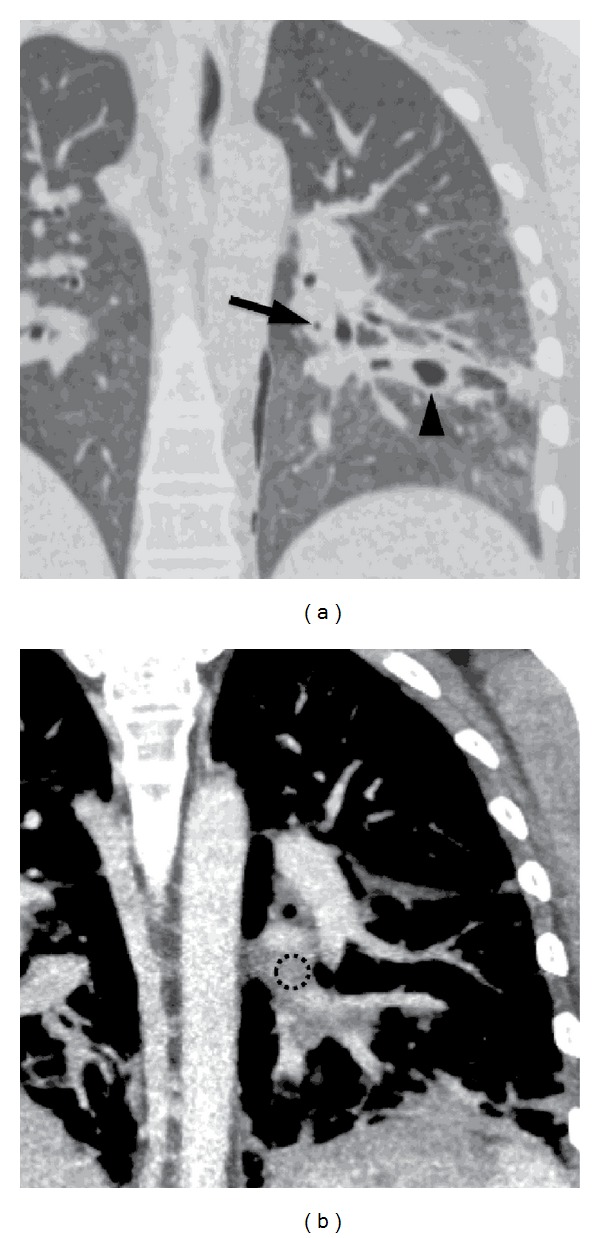
The lungs showing a focus of air in the pulmonary vein (arrow in (a)) and cavitary lesion (arrowhead). (b) is a CT with contrast performed earlier to show that the air is located in a pulmonary vein (dashed circle).

To workup her air embolism, a chest CT confirmed the small left pneumothorax, along with a 2.8 × 2.4 cm consolidative focus in the right upper lobe with an air crescent sign, favored as developing necrosis. This was thought secondary from an angioinvasive aspergillosis infection into her left pulmonary vein with demonstration of intravascular air (Figure 2). Repeated transthoracic echocardiogram revealed negative saline microcavitation study immediately and after Valsalva which was unrevealing for a cardiac shunt. An MRI brain on hospital day 57 confirmed multiple areas of restricted diffusion within the left MCA territory (Figure 1).

By the end of hospital day 57, her right side returned to baseline strength. She was able to speak in several word sentences appropriately but still had paraphasic errors and difficulty with repetition. By time of discharge on hospital day 68, speech therapy only noted minor paraphasic errors (95% accuracy with short story reading) with intact comprehension, repetition, and ability to communicate successfully in conversational speech. Her discharge NIH stroke scale was 1.

## 3. Discussion

This case demonstrates cerebral infarction from an air emboli originating from an angioinvasive pulmonary aspergillus source. Aspergillus species are ubiquitous in nature and inhalation of infectious conidia is a frequent event. In immunocompetent individuals, this does not result in clinical infection and tissue invasion is uncommon. In the setting of immunosuppression associated with therapy for hematologic malignancies, hematopoietic cell transplantation, or solid organ transplantation, the risk for infection is more substantial. One hallmark of pulmonary aspergillosis infection is vascular invasion with subsequent infarction and tissue necrosis. Once cavitary lesions are formed, they can produce air or be a conduit for air-filled lung tissue and, along with a pressure gradient, can either directly travel into the arterial system or indirectly through a venoarterial right to left shunt, with final progression into the cerebral vasculature [7].

This is the first reported case of stroke due to air embolism as a result of aspergillus infection, to our knowledge. The intravascular air emboli and the resulting brain parenchymal infarction are clearly demonstrated on CT and CT angiography. Chest imaging revealed pneumonia with cavitary lesions, compatible with aspergillus, which was confirmed with positive serum Galactomannan assays. Furthermore, intravascular air was also seen in a pulmonary vein adjacent to the cavitary lesion, implicating the cavitary lesion as the source of the air emboli. Interestingly, the patient did not develop hemoptysis, presumably due to subsequent thrombosis of the invaded vessel. Extensive investigation into other potential causes of air emboli was negative. This included echocardiography with microcavitation as well as cardiac MRI revealing no evidence of intracardiac or intrapulmonary shunting. The patient had not had any surgery, trauma, positive-pressure ventilation, or invasive procedures prior to this event.

For patients with air emboli, supportive care is of primary importance. Other considerations are prevention of further air emboli and removal of the embolized air. Patients with neurologic deficits should initiate HBOT as early as possible. There is a sharp decrease in the successful treatment of cerebral air emboli after a four- to five-hour delay [8]. In a retrospective cohort study of 86 patients with air embolism, those that initiated HBOT prior to 6 hours had better outcomes and improved recovery than those that initiated treatment later than 6 hours [9]. HBOT reduces the air bubble size and increases the arterial oxygen tension, though it is unclear which specific mechanism of action helps more and warrants further investigation [10].

## 4. Conclusion

This was a unique case of an immunocompromised patient with a pulmonary cavitary lesion caused by aspergillosis invading into a pulmonary vein and causing a cerebral air embolism. The patient suffered an acute ischemic stroke from the air embolism and was emergently taken for HBOT. Over the course of the next few days, her neurologic exam improved. In the case of cerebral air embolisms, the acute treatment option differs from the typical ischemic stroke pathway and the provider needs to consider emergent HBOT. This case report demonstrates the importance of remembering that atypical etiologies of acute ischemic strokes exist and other medical issues can contribute to an acute neurologic event.
